# Aging-related limit of exercise efficacy on motor decline

**DOI:** 10.1371/journal.pone.0188538

**Published:** 2017-11-27

**Authors:** Jennifer C. Arnold, Mark A. Cantu, Ella A. Kasanga, Vicki A. Nejtek, Evan V. Papa, Nicoleta Bugnariu, Michael F. Salvatore

**Affiliations:** 1 Department of Pharmacology, Toxicology, and Neuroscience, Louisiana State University Health Sciences Center, Shreveport, Louisiana, United States of America; 2 Department of Neurosurgery, New York University School of Medicine, New York, New York, United States of America; 3 Institute for Healthy Aging and Center for Neuroscience Discovery, University of North Texas Health Science Center, Fort Worth, Texas, United States of America; 4 Institute for Healthy Aging and Center for Alzheimer’s and Neurodegenerative Disease Research, University of North Texas Health Science Center, Fort Worth, Texas, United States of America; 5 Department of Physical & Occupational Therapy, Idaho State University–Meridian Health Science Center, Meridian, ID, United States of America; 6 School of Health Professions, University of North Texas Health Science Center, Fort Worth, Texas, United States of America; Northeastern Ohio Medical University, UNITED STATES

## Abstract

Identifying lifestyle strategies and allied neurobiological mechanisms that reduce aging-related motor impairment is imperative, given the accelerating number of retirees and increased life expectancy. A physically active lifestyle prior to old age can reduce risk of debilitating motor decline. However, if exercise is initiated after motor decline has begun in the lifespan, it is unknown if aging itself may impose a limit on exercise efficacy to decelerate further aging-related motor decline. In Brown-Norway/Fischer 344 F_1_ hybrid (BNF) rats, locomotor activity begins to decrease in middle age (12–18 months). One mechanism of aging-related motor decline may be decreased expression of GDNF family receptor, GFRα-1, which is decreased in substantia nigra (SN) between 12 and 30 months old. Moderate exercise, beginning at 18 months old, increases nigral GFRα-1 and tyrosine hydroxylase (TH) expression within 2 months. In aged rats, replenishing aging-related loss of GFRα-1 in SN increases TH in SN alone and locomotor activity. A moderate exercise regimen was initiated in sedentary male BNF rats in a longitudinal study to evaluate if exercise could attenuate aging-related motor decline when initiated at two different ages in the latter half of the lifespan (18 or 24 months old). Motor decline was reversed in the 18-, but not 24-month-old, cohort. However, exercise efficacy in the 18-month-old group was reduced as the rats reached 27 months old. GFRα-1 expression was not increased in either cohort. These studies suggest exercise can decelerate motor decline when begun in the latter half of the lifespan, but its efficacy may be limited by age of initiation. Decreased plasticity of GFRα-1 expression following exercise may limit its efficacy to reverse motor decline.

## Introduction

Parkinsonian signs are prevalent in the elderly, affecting ~15% of individuals by age 65 and 50% of individuals by age 80 [1–7]. Motor impairments like bradykinesia reduce the ability to perform activities of daily living and greatly compromise independent living capabilities [2,3] leading to increased risk of physical injury, cognitive impairment, dementia, and death [4]. The prevalence of aging-related locomotor impairments will increase with the doubling of the elderly population in the next 25 years. The number of US Citizens alone expected to reach age 100 will reach an unprecedented number by 2050 [8]. Thus, it is essential to determine the neurobiological basis of aging-related motor impairment and identify strategies that reduce risk and severity.

A physically active lifestyle can decrease risk of aging-related motor impairment [9–11]. However, the extent to which a controlled exercise regimen can attenuate decline in aging-related motor function, particularly after it has already begun in the latter half of the lifespan, is unknown. Furthermore, it is not known if aging may impose a ceiling upon the efficacy of a specific exercise regimen to attenuate motor decline. Study outcomes in Parkinson’s disease (PD) patients [12–15] and in PD models [16–19] suggest that exercise regimens can improve motor function, despite disability or impairments at exercise onset. Therefore, exercise could presumably be effective to attenuate or partially reverse aging-related motor decline after it has already begun. Some locomotor disabilities in aging humans may be reduced by exercise [20–22]. However, at least two limitations can affect the evaluation of the efficacy of a regimen to prevent further motor decline or partially restore it in aging. First, the metrics of exercise frequency, intensity, and duration are difficult to accurately establish from self-reporting [23]. Second, while these metrics can be controlled in preclinical exercise studies, these studies may not accommodate to the reality that supervised exercise in human studies is conducted for ~3 days/week [13,14,21], therefore leaving at least equal days within a time frame where exercise does not occur. This interspersion of rest within an exercise regimen should be accommodated in preclinical studies to optimize possible translation. Taken together, a controlled longitudinal exercise regimen that features exercise compliance to a regimen of fixed duration, intensity, and frequency, and accommodates for consistent rest periods, may provide a realistic and translatable outcome on the efficacy of a given exercise regimen on motor impairments related to aging and its underlying neurobiological mechanisms.

One possible mechanistic link between exercise impact on aging and locomotor function is glial cell-derived neurotrophic factor (GDNF) signaling. GDNF reduces motor impairment in aged rats and primates [24,25] Exercise can increase GDNF expression [26,27] or expression of its receptor, GFRα1 [28], and these increases are linked to increased dopamine (DA) content and tyrosine hydroxylase (TH) expression in substantia nigra (SN) [26,28]. However, aging may be a barrier for this exercise impact on GDNF signaling. Aging decreases GFRα1 expression, and its expression are correlated to TH expression in the nigrostriatal pathway [29]. However, increased GFRα1 receptor levels in the SN increases locomotor activity, with increased TH expression in SN alone of aged rats [30] suggesting that DA biosynthesis capacity and motor function may still be augmented at an advanced age. Therefore, augmentation of GDNF signaling through increased GFRα1 expression may be a mechanism by which the efficacy of an exercise regimen can attenuate or reverse aging-related motor decline.

In the Brown-Norway/Fisher 344 F_1_ hybrid (BNF) rat, aging-related locomotor decline begins between 12 and 18 months of age [31,32] Here, we determined the impact of a consistently applied exercise regimen on aging-related motor decline, initiated after locomotor decline is known to occur, in two cohorts where exercise was initiated at 18 or 24 months old. The intensity, frequency, and duration of exercise in both cohorts were identical. All exercise and non-exercise rats were trained and compliant to the regimen, without any negative reinforcement (i.e. footshock) [33]. Rest periods were also equally interspersed throughout the study, of approximately equal duration as exercise days, to reflect the reality of exercise frequency observed in human studies [34,35].

## Materials and methods

### Animals

Eighteen-month-old (n = 20, 447–646 g) and 24-month-old (n = 14, 520–718 g) male BNF rats were obtained from aging rodent colonies under the auspices of the National Institute on Aging (Charles River) and singly housed under controlled colony conditions with a 12-h reverse light-dark cycle (lights on at 1800 hours). The ages of these rats represent a time period after which aging-related motor decline is already underway [31,32,36,37]. Food and water were available *ad libitum* to rats throughout the study. Test subjects acclimated to the animal colony environment for at least 2 weeks prior to initiation of the study and were handled by the experimenter for 2–3 minutes per day for 1 week prior to any behavioral test. Treadmill and locomotor sessions were performed during the rats’ active (dark) portion of the light-dark cycle and completed before 16:30. Procedures were conducted in accordance with the Federal and Institutional Animal Care and Use Committee guidelines at LSU Health Sciences Center. Rats were euthanized immediately (within 1 hr) after conclusion of final locomotor assessment in the longitudinal study. The IACUC of the Louisiana State University Health Science Center of Shreveport approved the research conducted in this study. Euthanasia was conducted by decapitation immediately following application of isoflurane to render unconsciousness.

No rats died prior to meeting the humane endpoint. The longitudinal study time length was up to 8 months. Rat health was monitored, at minimum, on a weekly basis.

### Treadmill exercise regimen

The treadmill exercise regimen followed a footshock-free exercise training regimen [28,33]. The exercise regimen consists of three separate phases: pre-exercise, treadmill acclimation, and treadmill exercise. Treadmill exercise was conducted in rounds, with each round at an intensity of 8–9 m/min, a duration of 35 min, and a frequency of once per day for 12 consecutive days, followed by 14 days of rest, wherein the first 5 days of rest following exercise evaluated locomotor performance.

### Pre-exercise procedures

Body weights were recorded for all rats before and after each phase of the regimen. We have previously reported that two rounds of this exercise regimen produced weight loss, compared to non-exercised rats, of ~4% versus baseline body weight [33].

#### Baseline locomotor activity

Locomotor activity was assessed in open-field locomotor activity chambers (Opto Varimex 4 Animal Activity Monitoring System, Columbus Instruments, Columbus, OH, USA). Baseline locomotor capabilities prior to treadmill exercise were established to ensure the rats assigned to the exercise and non-exercise groups had non-significant differences in the locomotor parameters assessed at the start of the longitudinal study. To account for inherent possible daily variance in locomotor activity within each test subject [36], locomotor activity was assessed for 1h per day for 5 consecutive days as previously described [31,32,36] and the average value of the 5 assessments was used to establish each individual baseline. Locomotor capabilities were reported as total distance traveled (cm), horizontal activity (# of beam breaks), movement number (# of initiated movements), and movement speed (cm/sec). These parameters were chosen to represent overall locomotor capabilities because they enable evaluation of movement initiation capacity (movement number and horizontal activity), total distance, and movement speed (total distance divided by movement time).

### Treadmill acclimation phase

#### Acclimation phase 1

All treadmill acclimation and exercise sessions were conducted on a motorized rodent treadmill (Exer-4, Columbus Instruments, Columbus, OH, USA). During the first phase of treadmill acclimation, both exercise and non-exercise rats were placed on a stationary treadmill for 3 consecutive days. The duration of time on the stationary treadmill increased each day: 5 min (day 1), 7 min (day 2), and 10 min (day 3).

#### Acclimation phase 2

All test subjects, including the non-exercise group, were trained to exercise during a 7-day acclimation period to ensure that all rats in the study were physically able to participate in treadmill exercise. Rats were first trained to walk on the treadmill at low speeds for the first 3 sessions (5–7 m/min for 5–7 min) and more moderate speeds for the last 4 sessions (8–9 m/min; 8–10 min) of treadmill acclimation. With these acclimation procedures, we found >90% compliance to treadmill exercise without the use of footshock at any time [33].

#### Evaluating exercise compliance

Treadmill exercise scores, ranging from 1–4, were assigned after each acclimation and exercise session based on a test subject’s ability to adhere to the speed and duration of the session without assistance from the experimenter. An exercise score of 4 was assigned for rats that completed the session without any assistance from the experimenter, whereas a score of 1 denoted non-compliance to the regimen, and indicated that a test subject did not complete the session. A score of 3 indicated experimenter assistance was required for <25% of the total session time, and a score of 2 was assigned when test subjects required experimenter assistance for >25% of the session.

### Treadmill exercise phase

Treadmill exercise was conducted in rounds. A round consisted of 12 days of exercise followed by a 14-day rest period, wherein locomotor assessments were completed during the first 5 days of the rest period. Exercise sessions included a 5 minute warm-up period where rats exercised at 7–8 m/min before reaching 8–9 m/min. A total of 5 rounds of exercise and identical rest periods between the two age groups were conducted, with an additional 3 rounds in the 18-month-old group. The rest periods in the 3 additional rounds conducted for the 18-month old group varied between 4 weeks between rounds 5 and 6, ~2 weeks between rounds 6 and 7, and 1 week between rounds 7 and 8. The 24-month-old group only completed 5 rounds of the exercise regimen to avoid the possibility of other aging-related issues that could affect exercise ability or confound interpretation of locomotor outcomes. Notably, sarcopenia affects this rat strain after 30 months of age [38]. At the conclusion of the exercise regimens, there was a 2 month difference in age (27 vs 29 months old). Therefore, the age of the cohort beginning at 18 months old was similar to that of the 24-month-old cohort at the conclusion of exercise, essentially reaching equivalent age.

#### Post-exercise locomotor activity assessment

Locomotor activity was measured after every round of exercise in the same manner as previously described for baseline locomotor activity above. Locomotor activity sessions began the first day following the last exercise session of each round and continued for 5 consecutive days. The average value for all 5 locomotor activity sessions was calculated for each test subject to evaluate locomotor performance after each exercise round.

### Tissue collection and analysis

Rats were sacrificed by decapitation within an hour following the last locomotor activity session, representing the 5^th^ day after the last treadmill exercise session. Brain tissue was dissected from the SN, striatum, ventral tegmental area (VTA), and nucleus accumbens (NAc) as previously described [28,31,32,36] and stored at -80°C until processed for further analyses. Tissues were sonicated in ice-cold 0.1 M HClO_4_-EDTA and the supernatant was recovered after centrifugation to be analyzed for DA and DOPAC (as described in the subsequent section). The protein precipitate was sonicated in 1% SDS Tris EDTA solution and analyzed for total protein by the bicinchoninic acid method. We noted that protein recovery in the striatum, SN, and VTA was ~1.5-fold greater in tissues harvested from the 18-month-old group compared with the 24-month-old group. As normalization of DA tissue content is against total protein, this difference was reflected in overall lower DA per protein values in the 18-month-old group for these regions. For western blot detection, we used Clarity ^TM^ Western ECL Substrate (Cat. #: 170–5060, Bio-Rad Laboratories, USA). The primary antibody for detection of GFRα1 was rat GFRα1 Affinity Purified Goat IgG (0.5 μg/ml use dilution; Cat. #: AF 560, R & D Systems, Minneapolis, MN 55413, USA), using the secondary Rabbit anti-goat IgG (H+L)- HRP Conjugate (Cat #: 172–1034, Bio-Rad Laboratories, Inc, USA). For detection of TH, anti-Tyrosine Hydroxylase, rabbit, (1:1000 use dilution; Cat. #:AB152, Millipore, Temecula, CA 92590, USA) using the secondary Immun-Star Goat Anti-Rabbit (GAR)-HRP Conjugate #1705046, Bio-Rad Laboratories, USA).

The determination of GFRα1 was done by quantifying two bands representing the glycosylated and non-glycosylated insoluble form of GFRα1 (MW ~52–55 kDa) [29] using a nominal 40 μg total protein. Total TH protein was determined against a standard curve of TH standards loaded in a range of 0.5–4.0 ng total TH [28].

Images of the immunoreactive bands were captured using BioRad imager V3 ChemiDoc Touch System, and analyzed by Image Lab (BioRad) or Image J (NIH) software. The density of the immunoreactive bands was normalized against the relative total protein loaded from sample to sample by dividing the density value of GFRα1 or the ng of TH interpolated from the TH standard curve for each sample by the density value of the Ponceau-stained image of the associated sample lanes. All values are expressed as per total μg protein.

#### Determination of DA tissue content

Following tissue sonication in perchloric acid solution, supernatants were analyzed for content of DA by HPLC. A standard curve for DA and DOPAC ranging from 1.5 ng/ml to 800 ng/ml was generated using dopamine hydrochloride (CAS# 62-31-7), and 3,4-Dihydroxyphenylacetic acid (DOPAC) (CAS#102-32-9), both obtained from Sigma-Aldrich Corp (St. Louis, MO). The mobile phase contained 75 mM sodium dihyrdogen phosphate monohydrate, 1.7 mM 1-Octanesulfonic Acid sodium salt, 100 μL/L Triethylamine, 25 μM EDTA, 10% Acetonitrile, at a pH of 3.00, and was purchased from Fisher Scientific. The UHPLC pump (Ultimate 3000BM) was run at 0.6 mL/min isocratic gradient at ~255 Bar. The electrochemical (EC) detected was an Ultimate 3000RS electrochemical detector set to DC mode. Two cells were used in the EC detector, a 6020RS omni coulometric cell set at +300 mV to oxidize any contaminants in the mobile phase, and a 2-channel 6011RS ultra analytical cell set at -150 mV (Channel 1) and +220 mV (Channel 2). The analytical column was a BDS Hypersil (150mm X 3mm) C18 column with a particle size of 3μm. Samples and standards were placed in a WPS 3000TBRS autosampler maintained at 4°C during analysis. Injection volume for all standards and samples was 10 μL.

### Statistics

#### Body weight and relationship to locomotor activity

A Pearson r correlation analysis was used to evaluate if there was a relationship between baseline body weight and locomotor parameters. Body weights, weight loss, and percent change in body weight were all examined by two-way repeated measures ANOVA. A Tukey’s HSD post-hoc test was used to determine specific main effects of time during the regimen, whereas a Fisher’s LSD post-hoc test was used to determine specific group and time x group interaction effects.

#### Treadmill compliance

An unpaired two-tailed t-test was used to compare if differences in exercise training scores for treadmill acclimation existed between rats assigned to each group. A one-way repeated measures ANOVA determined if differences in treadmill exercise scores were evident after each round of exercise.

#### Aging and exercise effect determinations

To determine the effect of exercise upon aging-related locomotor decline, locomotor results were evaluated after each round of exercise for each test subject. A two-way repeated measures ANOVA was used to evaluate aging-related decline, and if exercise intervention affected locomotor activity decline due to aging, by evidence of interaction between the age versus exercise. A post-hoc Fisher’s LSD test was used to determine exercise effects on aging at each time point of evaluation for each round of exercise regimen for the 2 ages of exercise intervention.

The Grubb’s outlier test detected any outliers (as a percent of the baseline) at each round of analysis. If outliers were detected, the group average for that outlier replaced the percent of baseline value for that individual rat at that round. Outliers were detected in at least one round, but not more than three of the eight rounds, for a total of three of the 20 rats (2 of 10 from the non-exercise group and 1 of 10 from the exercise group) used in the 18-month-old study. In the 24-month-old group, one outlier was detected for 1 round during the regimen for one rat in the non-exercise group. An unpaired two-tailed t-test determined if significant differences existed between exercise and non-exercise rats for neurochemical measures. Significance for all statistical tests was set at *p*<0.05.

## Results

### Impact of long-term treadmill exercise on body weight

Body weights ranged from 554–606 grams for both age groups and were not significantly different between non-exercise and exercise rats in either age group at the start of the exercise acclimation. In the 18-month-old group, body weight decreased in the second acclimation period compared to the first acclimation period in both groups (S1 Fig). Exercise and non-exercise rats in the 24-month-old group also lost significantly more weight during the second acclimation period compared to the first acclimation period in both groups (S1 Fig). These results are consistent with our previously reported findings [33].

In the 18-month-old group, body weights significantly decreased in both exercise and non-exercise groups during the exercise regimen (S1C Fig), with the exercise group losing more weight after rounds 3 and 7. Similarly, in the 24-month-old group, body weights also decreased during the exercise regimen in both exercise and non-exercise groups (S1D Fig). Exercise rats lost more weight after round 3 (*p*<0.05).

A Pearson correlation analysis showed no significant relationship between baseline body weight against any of the 5 locomotor parameters at baseline or after exercise in the 18-month-old (Tables 1 and 2) or 24-month-old group (Tables 3 and 4). This lack of association of body weight with motor function has been previously reported [31,32].

**Table 1:** Correlations of body weight & locomotor parameters at baseline, 18-month-old group.

Baseline Body Weight versus Baseline Locomotor Parameter: Exercise & Non-Exercise Groups				
	Non-Exercise		Exercise	
Locomotor Parameter	Correlation r Value	*p*-Value	Correlation r Value	*p*-Value
Total Distance	-0.184	0.61	0.116	0.75
Horizontal Activity	-0.0324	0.93	0.0494	0.89
Movement Number	-0.108	0.77	0.168	0.64
Movement Time	-0.0427	0.91	-0.263	0.46
Movement Speed	-0.0497	0.14	0.261	0.47

**Table 2:** Correlations of body weight & locomotor parameters after exercise, 18-month-old group.

Post-Exercise 8 Body Weight versus Post-Exercise 8 Locomotor Parameter: Exercise & Non-Exercise Groups				
	Non-Exercise		Exercise	
Locomotor Parameter	Correlation r Value	*p*-Value	Correlation r Value	*p*-Value
Total Distance	0.333	0.35	0.532	0.11
Horizontal Activity	0.325	0.36	0.542	0.11
Movement Number	0.380	0.28	0.541	0.11
Movement Time	0.155	0.67	0.091	0.70
Movement Speed	0.362	0.30	0.433	0.21

**Table 3:** Correlations of body weight & locomotor parameters at baseline, 24-month-old group.

Baseline Body Weight versus Baseline Locomotor Parameter: Exercise & Non-Exercise Groups				
	Non-Exercise		Exercise	
Locomotor Parameter	Correlation r Value	*p*-Value	Correlation r Value	*p*-Value
Total Distance	-0.707	0.08	0.568	0.18
Horizontal Activity	-0.583	0.17	0.651	0.11
Movement Number	-0.488	0.27	0.640	0.12
Movement Time	-0.631	0.13	0.616	0.14
Movement Speed	-0.544	0.21	0.031	0.95

**Table 4:** Correlations of body weight & locomotor parameters after exercise, 24-month-old group.

Post-Exercise 5 Body Weight versus Post-Exercise 5 Locomotor Parameter: Exercise & Non-Exercise Groups				
	Non-Exercise		Exercise	
Locomotor Parameter	Correlation r Value	*p*-Value	Correlation r Value	*p*-Value
Total Distance	-0.418	0.35	0.495	0.26
Horizontal Activity	-0.281	0.34	0.557	0.19
Movement Number	0.360	0.43	0.521	0.23
Movement Time	-0.250	0.59	0.494	0.26
Movement Speed	-0.505	0.25	-0.0108	0.98

### Exercise compliance in aging rats

**Fig 1:**
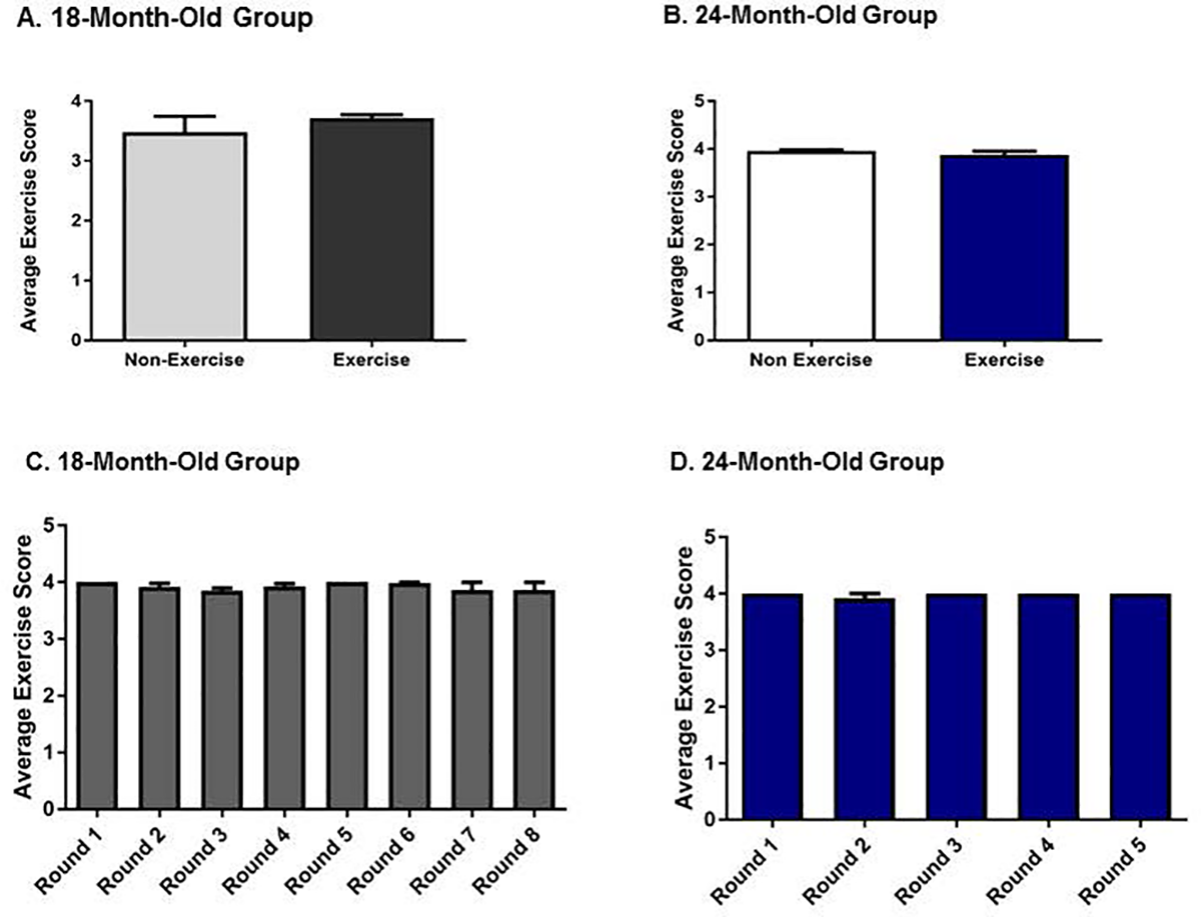
Treadmill exercise compliance scores during treadmill acclimation and exercise. **A. Treadmill acclimation scores in 18-month-old rats.** Average exercise scores were not significantly different between groups (t = 0.85, *p* = 0.41). **B. Treadmill acclimation scores in 24-month-old rats**. Average exercise scores were not significantly different between groups (t = 0.98, *p* = 0.35). **C. Treadmill exercise scores in 18-month-old exercise group.** Average exercise scores were not significantly different between any round of exercise (F_(1,9)_ = 1.00, *p* = 0.34). **D. Treadmill exercise scores in 24-month-old exercise group**. Average exercise scores were not significantly different between any round of exercise (F_(1,6)_ = 1.00, *p* = 0.36).

Consistent with our previously reported findings [27,32] no significant differences in treadmill exercise compliance were observed between the exercise and non-exercise groups during treadmill acclimation for either age group (Fig 1A and 1B). All test subjects in the exercise group were compliant to treadmill exercise for the duration of the study (Fig 1C and 1D).

### Impact of treadmill exercise initiation on aging-related locomotor decline at 18- versus 24-months old

**Fig 2:**
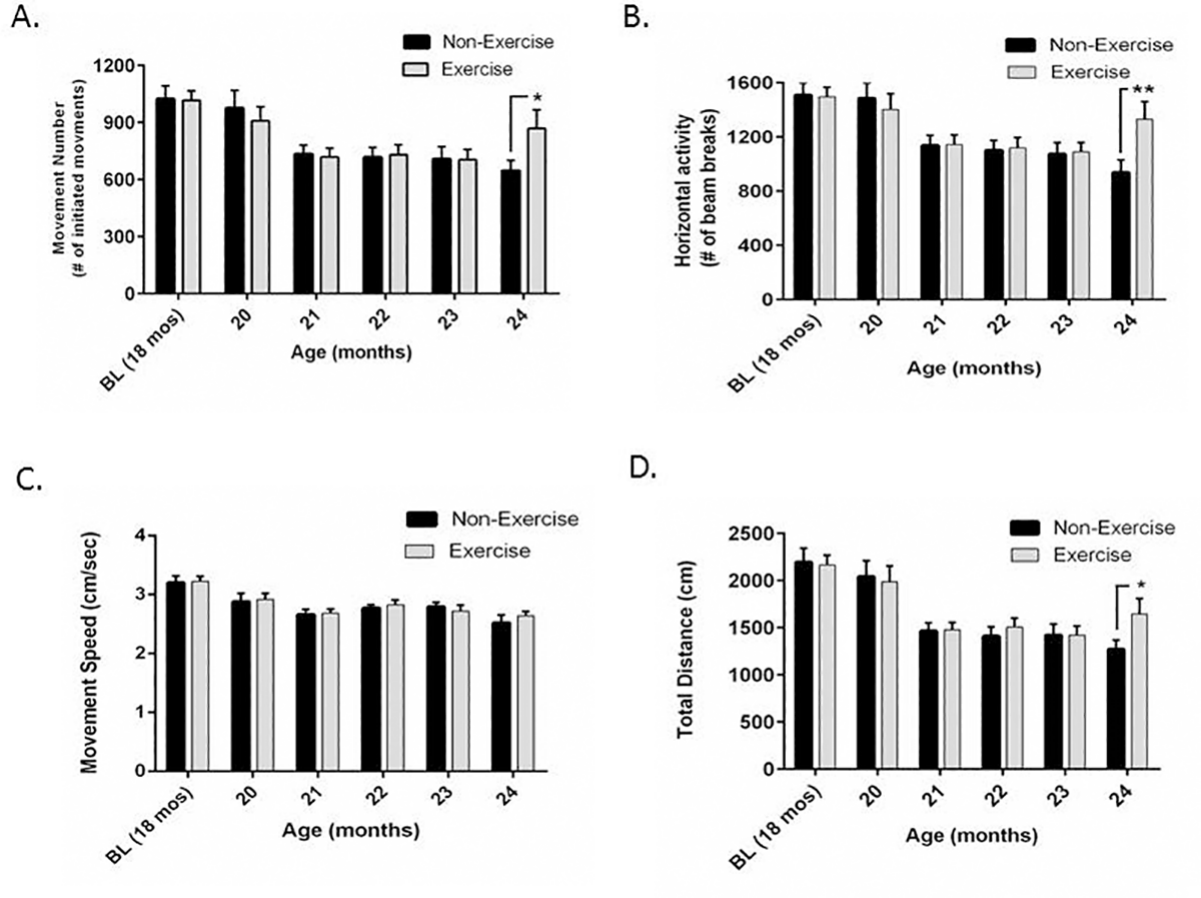
Impact of exercise initiation at 18-months-old on aging-related motor decline. **A. Movement number.** Treadmill exercise attenuated aging-related decline in movement number. Aging produced a significant decrease in movement number (F_(5,90)_ = 18.67, *p<*0.0001). There was significant interaction between aging and exercise (F_(5,90)_ = 2.69, *p* = 0.026). After 5 rounds of the exercise regimen, there was a significant increase in movement number in the exercise group (t = 2.44, *p = 0.016). **B. Horizontal Activity.** Treadmill exercise attenuated aging-related decline in horizontal activity. Aging produced a significant decrease on horizontal activity (F_(5,90)_ = 20.18, *p<*0.0001). There was significant interaction between aging and exercise (F_(5,90)_ = 4.13, *p* = 0.002). After 5 rounds of the exercise regimen, there was a significant increase in movement number in the exercise group (t = 3.04, **p = 0.003). **C. Movement speed.** Treadmill exercise did not attenuate aging-related decline in movement speed. Aging produced a significant decrease on movement speed (F_(5,90)_ = 14.12, *p<*0.0001). There was no significant interaction between aging and exercise on movement speed (F_(5,90)_ = 0.27, *p* = 0.93). There was no significant difference in movement speed associated with the exercise regimen at any of the 5 rounds. **D. Total distance.** Treadmill exercise attenuated aging-related decline in total distance. Aging produced a significant decrease on total distance (F_(5,90)_ = 29.95, *p<*0.0001). No interaction between aging and exercise was indicated (F_(5,90)_ = 1.69, *p* = 0.15). Exercise attenuated aging-related decline in total distance after round 5 (t = 2.16, *p<0.05). For all parameters, there was highly significant matching of test subjects (Movement number, F_(18,90)_ = 8.20, *p<*0.0001; Horizontal activity, F_(18,90)_ = 9.47, *p<*0.0001; Movement speed, F_(18,90)_ = 3.05, p = 0.0003); Total distance, F_(18,90)_ = 6.95, *p<*0.0001).

In 18-month-old rats, there was an effect of aging in both groups on the movement parameters evaluated. There was also a significant interaction between exercise and aging, indicating exercise affected the trajectory of aging-related decline in movement frequency as the number of whole body movements (ambulatory counts and horizontal activity) was significantly greater in the exercise group compared to the non-exercise group by round 5 (Fig 2A and 2B). Aging-related decline in movement speed was not affected by the exercise regimen (Fig 2C). Total distance was increased in the exercise cohort by the 5^th^ round of the longitudinal exercise regimen, although no significant interaction was observed between exercise and the aging-related decline. Significant differences in movement number, horizontal activity, and total distance were evident between the two groups as they reached 24 months of age (Fig 2A, 2B and 2D). These results indicate that consistent exercise between 18 and 24 months of age may reverse or partially restore aging-related loss of locomotor activity.

**Fig 3:**
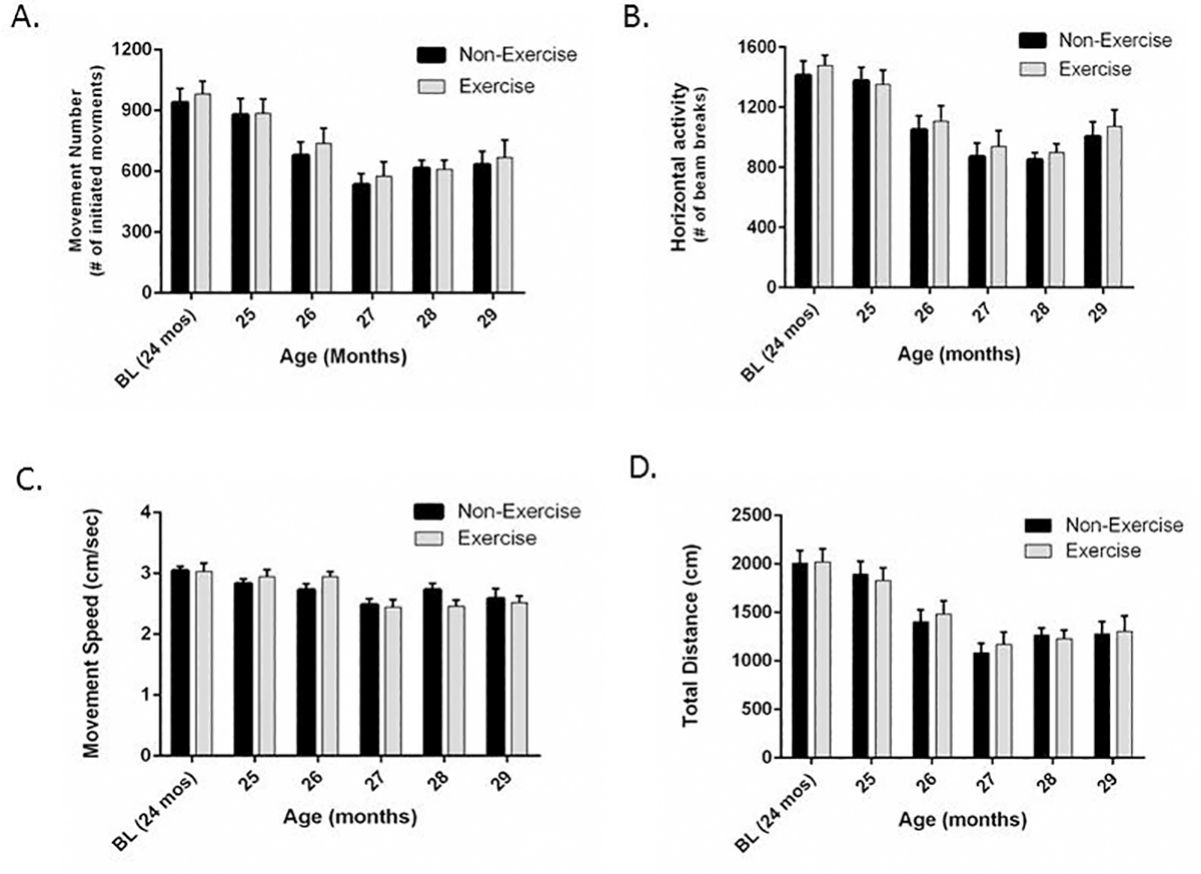
Impact of exercise initiation at 24-months-old on aging-related motor decline. **A. Movement number.** Treadmill exercise over 5 rounds did not attenuate aging-related decline in movement number. Aging produced a significant decrease in movement number (F_(5,60)_ = 29.49, *p<*0.0001). Exercise impact on movement number over 5 rounds was not significant between the two groups (F_(1,12)_ = 0.15, *p =* 0.71), and there was no significant interaction between aging and exercise (F_(5,60)_ = 0.16, *p* = 0.98). **B. Horizontal Activity.** Treadmill exercise over 5 rounds did not attenuate aging-related decline in horizontal activity. Aging produced a significant decrease on horizontal activity (F_(5,60)_ = 34.76, *p<*0.0001). Exercise impact on horizontal activity was not significant between the two groups (F_(1,12)_ = 0.21, *p =* 0.66), and there was no significant interaction between aging and exercise (F_(5,60)_ = 0.20, *p* = 0.96). **C. Movement speed.** Treadmill exercise over 5 rounds did not attenuate aging-related decline in movement speed. Aging produced a significant decrease on movement speed (F_(5,60)_ = 17.72, *p<*0.0001). Exercise impact on movement speed over 5 rounds was not significant between the two groups (F_(1,12)_ = 0.01, *p =* 0.92), but there was significant interaction between aging and exercise (F_(5,60)_ = 2.46, *p* = 0.04). **D. Total distance.** Treadmill exercise over 5 rounds did not attenuate aging-related decline in total distance. Aging produced a significant decrease on total distance (F_(5,60)_ = 38.91, *p<*0.0001). Exercise impact on total distance was not significant between the two groups (F_(1,12)_ = 0.01, *p =* 0.90), and there was no significant interaction between aging and exercise (F_(5,60)_ = 0.28, *p* = 0.92). For all parameters, there was highly significant matching of test subjects (Movement Number, F_(12,60)_ = 10.43, *p<*0.0001; Horizontal Activity, F_(12,60)_ = 9.94, *p<*0.0001; Total Distance, F_(12,60)_ = 8.90, *p<*0.0001; Movement Speed, F_(12,60)_ = 7.81, *p<*0.0001).

In contrast, when the same treadmill exercise regimen was initiated in the 24-month-old cohort, the exercise group did not show any difference in movement frequency parameters. A two-way repeated measures ANOVA analysis revealed no significant interaction between exercise and aging for the parameters associated with movement initiation, indicating that the exercise initiation at 24 months of age did not alter the trajectory of aging-related decline in locomotor activity when initiated 6 months later in the lifespan (as compared to initiation at 18 months of age) (Fig 3A, 3B and 3D). However, there was a significant interaction between exercise and aging for movement speed (Fig 3C).

### Impact of exercise continuation in 18-month-old rats

**Fig 4:**
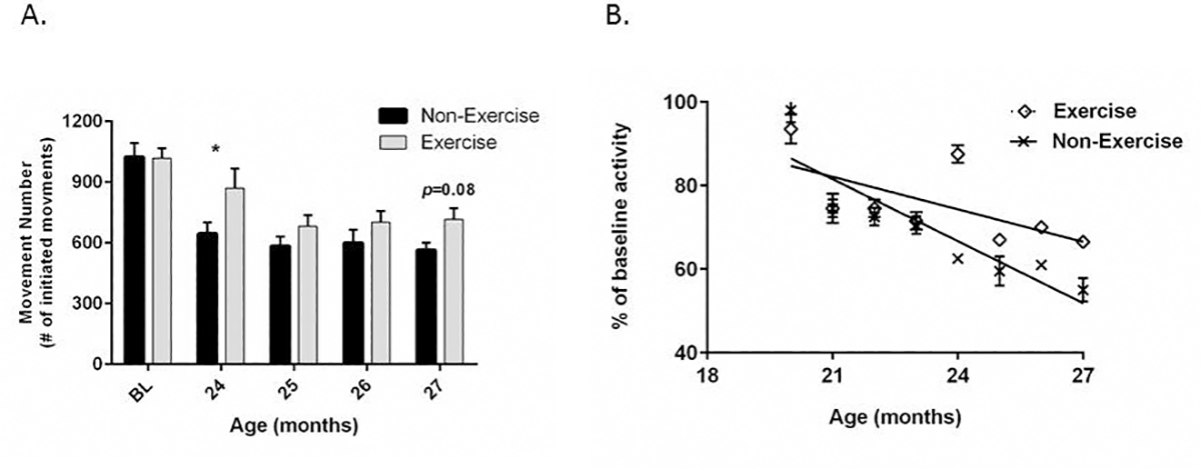
Impact of 8 months of exercise on aging-related decline in locomotor activity from 18–27 months old. **A. Movement number.** The increase in movement number from the exercise regimen at 24 months of age was not observed in the subsequent 2 rounds, but a trend toward increased locomotor activity was seen at when the rats reached 27 months of age 2 way Repeated Measures ANOVA results for all 8 rounds of exercise showed highly significant interaction (F_(8,144)_ = 2.76, *p*<0.01) between aging effects (F_(8,144)_ = 23.30, *p*<0.0001) and exercise. (round by round outcome; round 5 (t = 2.44, *p = 0.016), round 6 (t = 1.19, ns), round 7 (t = 1.16, ns), round 8 (t = 1.75, ns). **B.** Linear regression analysis of aging-related decreases in locomotor activity (combined movement number and horizontal activity results) against baseline in non-exercise vs exercise groups out to 27 months of age. There was significant deviation from zero in the non-exercise group (x) (F_(1,14)_ = 52.91, *p*<0.0001; R^2^ = 0.791) and in the exercise group (◊) (F_(1,14)_ = 9.71, *p* = 0.007; R^2^ = 0.410). There was a significant difference in the slope of aging-related decline in movement frequency between the non-exercise (-4.95 ±0.68) and exercise groups (-2.60 ± 0.83), (F_(1,28)_ = 4.78, *p*<0.05).

The longitudinal exercise regimen continued an additional 2–3 months in the 18-month-old group. The significant increase in locomotor activity in the exercise group observed earlier at 5 rounds was no longer observed at any of the 3 additional rounds, although there was a trend toward an increase in movement number at the 3^rd^ additional round (*p =* 0.08) (Fig 4A).

The locomotor parameters of movement initiation (movement number and horizontal activity) were evaluated together by analyzing the aging-related departure from baseline performance in linear regression analysis. There was a significant difference in the slope of the aging-related decline in movement frequency in the exercise group, indicating that exercise decelerated aging-related decline in movement frequency (Fig 4B).

**Fig 5:**
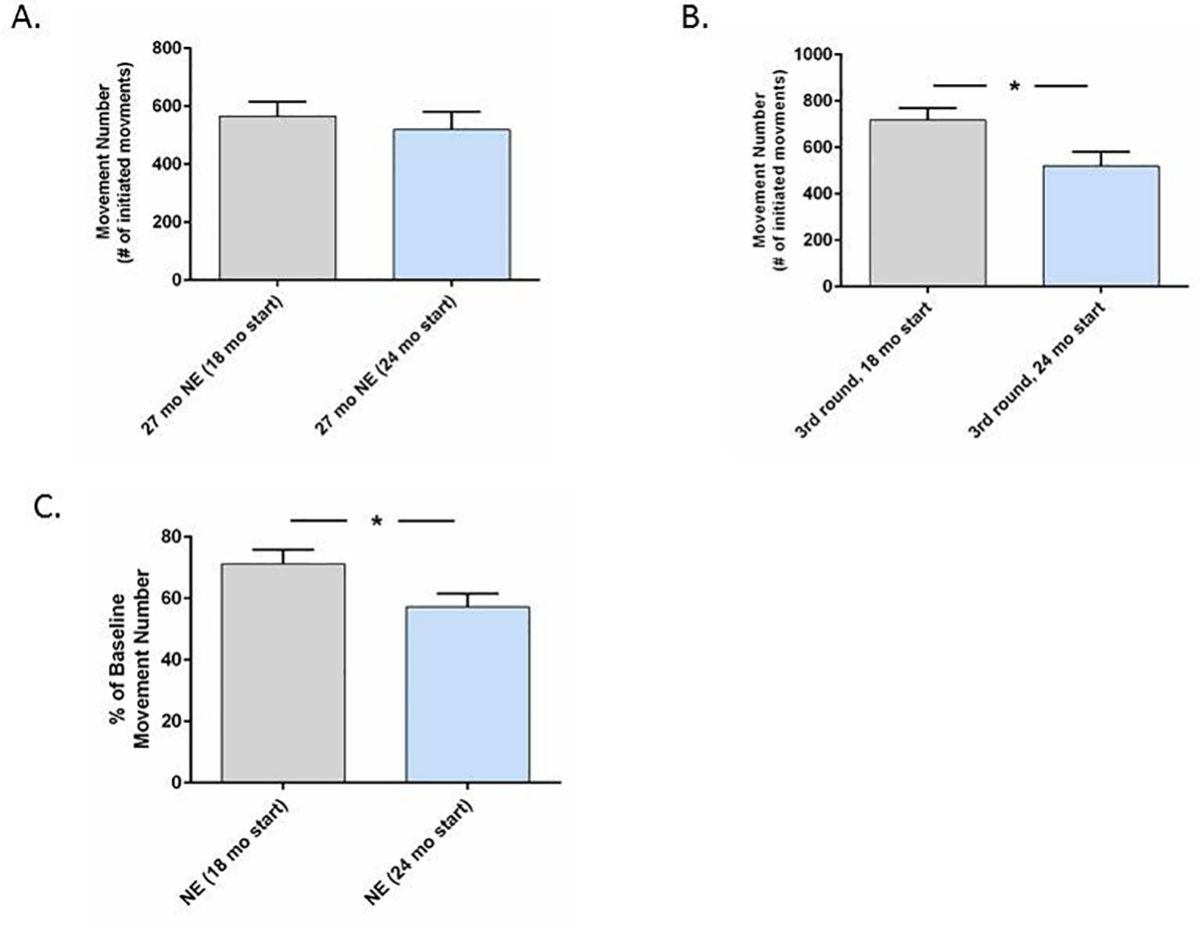
Comparison of locomotor activity in the non-exercise groups between 18-month- and 24-month-old groups. **A. 27 months of age in both groups.** Movement number in non-exercise groups was not significantly different between the 18- and 24-month-old groups when the cohorts were 27 months old (*t* = 0.58, *p* = 0.57). **B. After 3 rounds of the regimen.** Movement number was greater in the 18-month-old group (corresponding to 22 months of age versus 27 months of age in the 24-month-old group) (*t* = 2.48, *p<0.05). **C. Percent change in movement number against baseline activity after 3 rounds.** The percent decrease in movement number was significantly greater in the 24 month-old group (*t* = 2.14, *p<0.05).

There were similar activity levels between the two age groups at the start of their respective studies (Figs 2 and 3). We determined if this similarity would potentially confound the interpretation of exercise effects against the age of initiation. We therefore analyzed activity levels in the non-exercise groups in two analyses; 1) when rats were of equal age during the two respective studies, and 2) after an equal number of exercise regimen rounds. There was no significant difference in movement number between the two age groups when they reached 27 months of age (last round of 8 for the 18-month-old group and 3^rd^ round for the 24-month-old group) (Fig 5A), indicating comparable movement in the non-exercise controls between the two cohorts when they reached the same age in their respective studies. Second, expected differences in locomotor activity between the two age groups in the non-exercise controls was evident by round 3 of the regimen, with greater movement number in the 18-month-old cohort (age 22 months) versus the 24-month-old cohort (age 27 months) (Fig 5B). Finally, the rate of locomotor decline against baseline was significantly greater in the 24-month-old cohort by round 3 (Fig 5C). Taken together, these results indicate that the 24-month-old cohort exhibited expected differences in aging-related decline during the course of respective studies, despite similar activity levels versus the 18 month old group at the baseline assessment.

### Impact of exercise on GFRα1, TH expression, and DA tissue content

**Fig 6:**
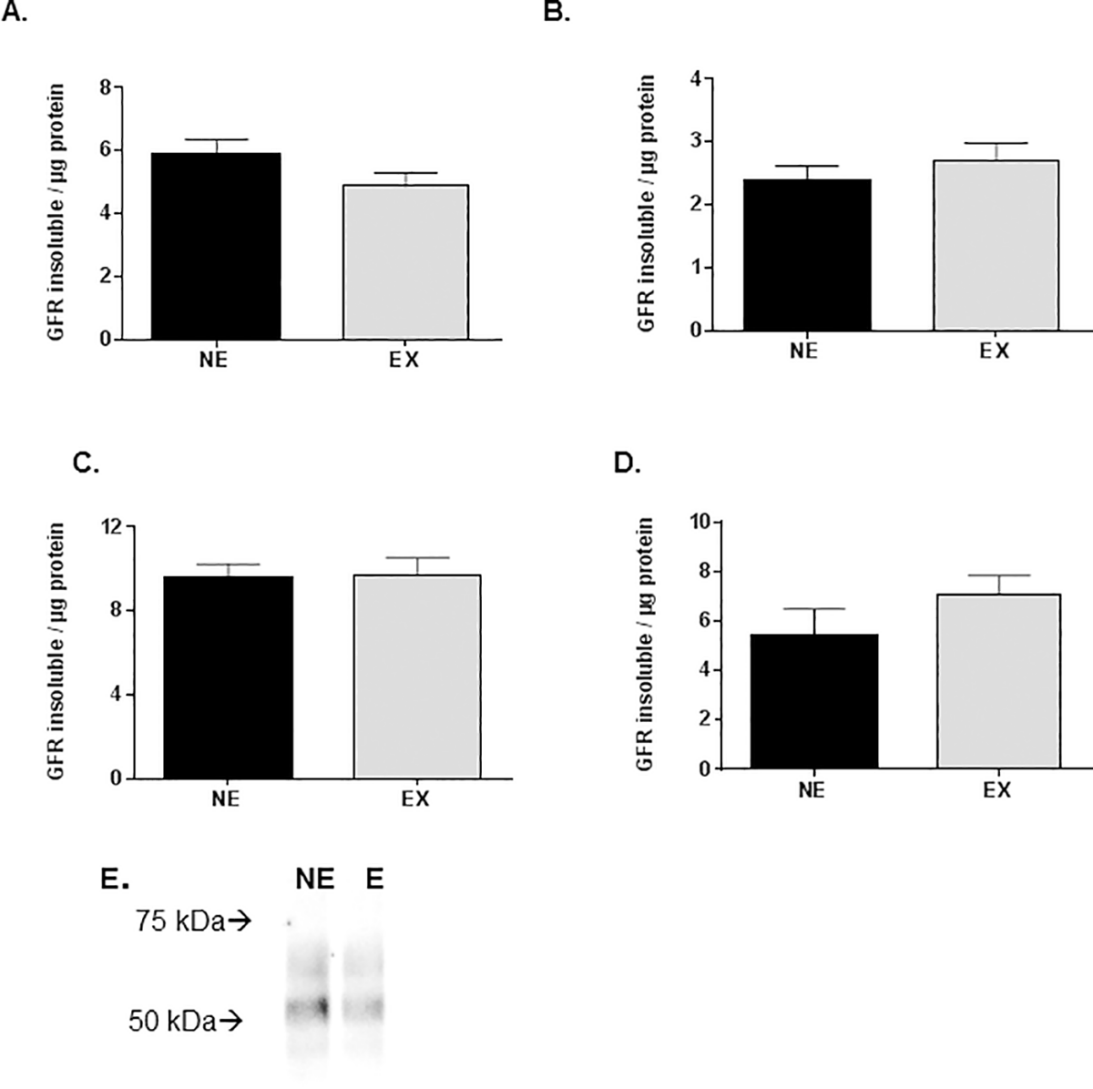
**Expression of GFRα1 in 18 month (A,B) and 24 month (C,D) cohorts. A. Striatum**. Expression of the insoluble form of the GFRα1 receptor was not significantly different between the exercise and non-exercise groups (t = 1.54, df = 16, ns). **B. SN**. Expression of the insoluble form of the GFRα1 receptor was not significantly different between the exercise and non-exercise groups (t = 0.82, df = 15, ns). **C. Striatum**. Expression of the insoluble form of the GFRα1 receptor was not significantly different between the exercise and non-exercise groups (t = 0.13, df = 11, ns). **D. SN**. Expression of the insoluble form of the GFRα1 receptor was not significantly different between the exercise and non-exercise groups (t = 1.26, df = 10, ns). Results are expressed as arbitrary units of immunoreactivity normalized against total protein recovered in each lane, nominal 30 μg protein load. **E. Representative blot image of GFRα1 (insoluble form) from 18-month-old NE and E subject**.

The GFRα1 receptor is biologically active in two forms, a GPI-linked (insoluble) and soluble form [39] and both forms are present and detected in the rat brain, including striatum and SN [28,29,40]. GFRα1 expression decreases in the SN aging [28], but the treadmill exercise regimen used in this study increases expression of the insoluble form of GFRα1 in SN after 2 rounds of exercise when exercise is initiated at 18 months old and completed by 20 months old [28]. However, after 8 rounds of exercise in this study, we did not find any significant effect of the exercise regimen in the 18-month-old cohort (Fig 6A and 6B). GFRα1 expression was also unaffected by exercise in the 24-month-old cohort (Fig 6C and 6D).

**Fig 7:**
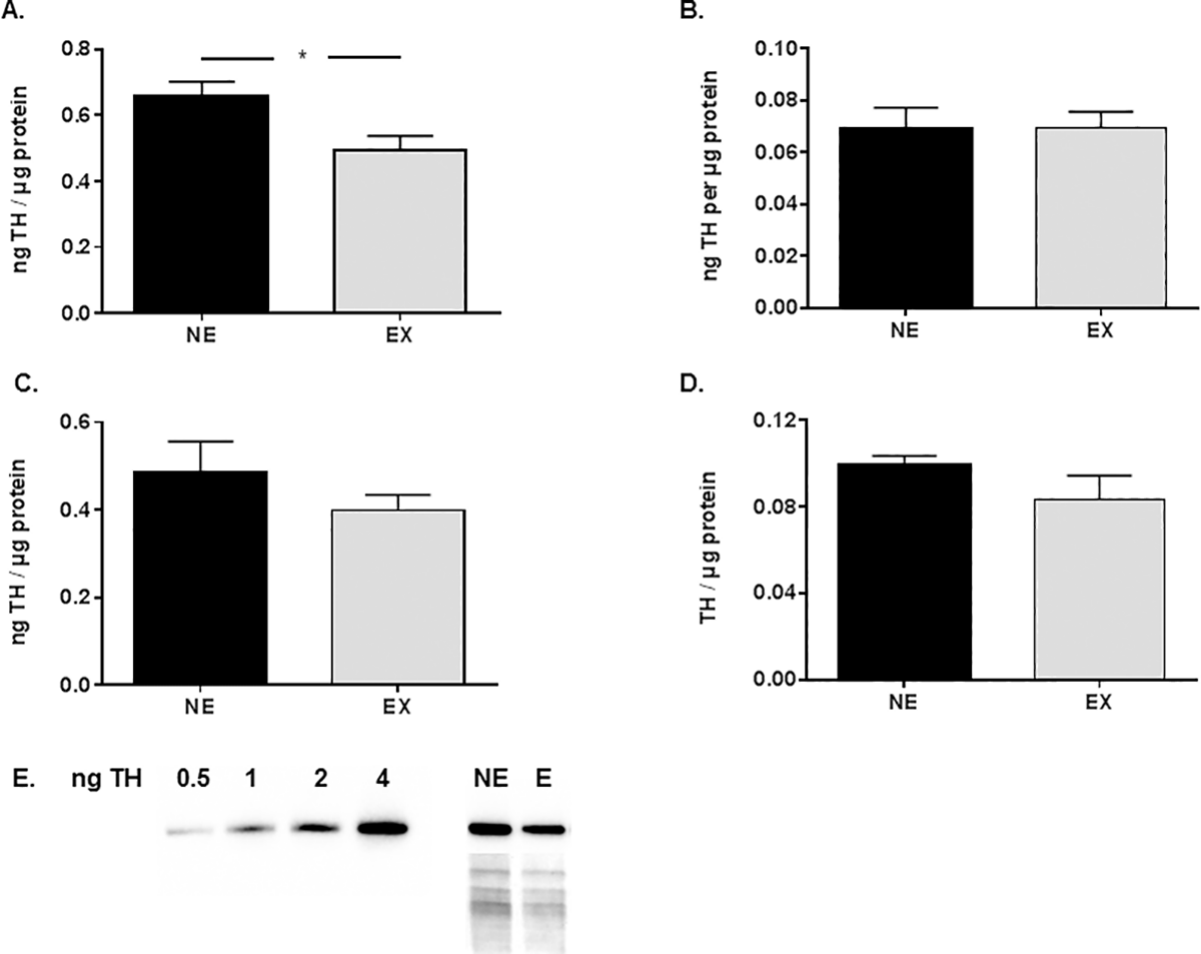
**Expression of TH protein in 18 month (A,B) and 24 month (C,D) cohorts. A. Striatum**. Expression of TH protein was significantly decreased in the exercise group (t = 2.56, df = 16, *p<0.05). **B. SN**. Expression of TH protein was not significantly different between the exercise and non-exercise groups (t = 0.03, df = 17, ns). **C. Striatum**. Expression of TH protein was not significantly different between the exercise and non-exercise groups (t = 1.09, df = 12, ns). **D. SN**. Expression of TH protein was not significantly different between the exercise and non-exercise groups (t = 1.25, df = 11, ns). All results are expressed as ng total TH protein per total μg protein. **E. Representative blot of TH assay result from striatum.** TH standard was loaded from 0.5 to 4 ng. A non-exercise (NE) and exercise (E) result is shown representing the overall ~25% decrease in TH expression in the exercise group. Below the two representative samples is the associated Ponceau stain for the area of the lane where TH is resolved between 50 and 75 kDa.

Expression of TH protein was differentially affected by the exercise regimen at its conclusion in the 18-month-old cohort, as we observed a significant decrease in TH expression in the striatum, without effect in the SN (Fig 7A and 7B). No differences in TH expression by exercise were observed in the 24-month-old cohort (Fig 7C and 7D).

There was no significant difference in striatal or nigral DA tissue content in either age group at the completion of the study (S2 Fig). However, we did evaluate DA tissue content in the VTA and NAc. There was a noted trend (*p* = 0.06) in DA tissue content in the VTA in the 18-month-old cohort (S2 Fig). The DA metabolite, dihydroxyphenyacetic acid (DOPAC), or the DOPAC/DA ratio, was not significantly different in any of the 4 regions in either age group (data not shown).

## Discussion

To assess the neurobiological basis of exercise-related impact on aging-related impairments, a systematic evaluation featuring control of exercise parameters and the timing of evaluations over a significant portion of the lifespan is vital. Our study represents a rigorous step in this direction, by administering a longitudinal exercise regimen at two different time periods in the latter half of the lifespan with fixed parameters of intensity, duration, and frequency. Notably, the exercise intervention in this study began after locomotor impairment likely has already occurred, 6 months prior to the earliest intervention at 18 months old [31,32]. Consistent rest periods also were introduced into this regimen. Controlled exercise studies in humans have participants exercise for roughly half the number of days over the entire course of a study (e.g. 3x/week). Our longitudinal exercise regimen reflects this practice in human subjects research, with equal exercise and rest days over the course of the study. We note that we did not intersperse the rest periods exactly as done in human studies because we needed to evaluate locomotor activity at specific and equal exercise intervals, without the possible confound of exercise-related fatigue affecting locomotor activity results. With these practical constraints, our longitudinal studies reveal that consistent, moderate treadmill exercise initiated in previously sedentary rats at an advanced middle age (18 months old) may reverse aging-related decreases in locomotor activity (Fig 2) and attenuate further aging-related decline into advanced age (Fig 4B). Second, these results also indicate the possibility that the age of exercise initiation can influence the efficacy of exercise to decelerate aging-related motor decline. The same exercise regimen did not attenuate aging-related locomotor decline when initiated 6 months later in the lifespan (24 months old) (Fig 3). This differential outcome does not necessarily indicate that exercise would not attenuate aging-related motor decline if initiated at 24 months of age. Rather, these results suggest a starting point in which the variables of exercise (intensity, duration, or frequency) and rest periods could be altered to possibly reduce aging-related motor decline when exercise begins at a more advanced age.

The outcome of this study indicates the possibility that previously sedentary individuals of an age approaching retirement may actually improve motor performance if beginning consistent exercise as a lifestyle habit in advanced middle-age. Furthermore, such a motor benefit may be realized even with interspersion of rest periods of equal duration as exercise. The sedentary lifestyle in humans dominates the waking hours in older adults, as up to 67% of adults are sedentary for 8.5 h/day [41], which portends to increased risk of locomotor impairment and adverse quality of life issues. Recent human studies strongly support that greater physical activity in middle age (30s-50s) reduces vulnerability to aging-related locomotor impairment and risk of mortality [10,11,42]. In fact, physical activity levels above 65 years old inversely correlate to the need for utilization of health care services [43]. Our results in the 18-month-old group suggest that with consistent exercise and equal rest period time, the negative impact of a previously sedentary lifestyle on aging-related locomotor impairment can be at least partially averted. A nearly life-long (5 to 23 months of age) treadmill exercise paradigm on locomotor behaviors with double the intensity (20 meters/min) used in our study attenuated aging-related decreases in spontaneous activity beginning at 12 months of age and continued until 23 months [44,45]. Our findings suggest that exercise can be initiated much later in the lifespan and still prove effective in reducing aging-related motor decline. However, our findings also indicate that there may be an aging-related limit to exercise efficacy, as shown with intervention at 24 months of age, given equal application of the exercise parameters of frequency, duration, and intensity.

The lack of exercise effect on motor function in the 24-month-old cohort does not unequivocally indicate that any exercise regimen would not attenuate aging-related motor decline. In fact in humans, balance-, resistance-, or strength-based training can be effective exercise-related approaches to improve motor impairments [46–48]. From the treadmill-based approach, one variable that may be altered and evaluated for possible exercise benefit in older rats is increasing the frequency of exercise. Our compliance scores and experience with aged rats strongly suggest that the limit of exercise intensity and duration on the treadmill were reached [28,33]. We note there is a high level of compliance with this treadmill speed in the absence of footshock application at any time. Imposing footshock certainly would confound interpretation of catecholamine-influenced behavior (like locomotor function), given its known impact [49,50]. Furthermore, the duration of 35 min is achievable, but longer periods of exercise for rats of this age may not be realized without undue application of negative reinforcement. The final 3 rounds of exercise in the 18-month-old cohort indicate that shortening of the rest period may yield significant effects, despite the advanced age. The rest period in this cohort was shortened by half between the 7^th^ and 8^th^ rounds of the regimen, when the rats reached 27 months of age. Notably, locomotor activity values were comparable at that time in the non-exercise groups between the two age groups (Fig 5A). We also point out that our assay of evaluating locomotor decline via locomotor activity was limited; and some exercise effects on other motor function may have been missed [51], although Seldeen also reported no effect of their regimen on total distance, similar to the outcome in the older intervention group of our study. Furthermore, fatigue is an issue in aging [52], affecting performance in functional tasks related to locomotor function [53]. The possibility of greater fatigue vulnerability in the 24-month-group may have negated observations of increased locomotor capacity. Still, it is also possible that exercise intervention may not be effective if initiated with an additional 6 months of sedentary activity in the lifespan prior to exercise initiation. The outcomes of exercise intervention in the elderly are mixed [54–56]. However, studies in those with PD have shown efficacy, particularly those with lower than average baseline performance [14].

The locomotor effect in the 18-month-old exercise group was not likely related to a calorie restriction effect, given that weight loss in the exercise group (2.4%) was far less than calorie restricted rats (15%), wherein similar locomotor effects were observed [31,32]. Differences in weight between the respective control groups (non-exercise and *ad libitum* fed) were much greater in the calorie restriction study (<1% vs. 24%, respectively). Therefore, differences in maintaining locomotor functions against aging, as seen in this study, are likely not attributable to weight loss akin to calorie restriction. Most important, no relationship of weight versus locomotor activity was seen in this study (Tables 1 and 2) or the aforementioned calorie restriction study.

Exercise produced a significant decrease in TH expression in the striatum in the 18-month-old cohort. This result may be unexpected given that loss of TH is also seen in aging or in PD, although the degree of loss in aging is far less than in PD [57,58]. We recently reported that 30% calorie restriction also reduced striatal TH expression, despite that calorie restriction prevented aging-related motor decline from 12 to 18 months old [32]. We speculate this decrease may be related to an exercise-related increase in GDNF expression [26,27]. GDNF decreases TH expression in striatum in the aged rat [59]. Calorie restriction also increases GDNF expression [60]. Calorie restriction, like exercise, also has been recently shown to prevent aging-related motor decline, notably in conjunction with decreased striatal TH expression [32]. The lack of effect of exercise on striatal TH in the 24-month-old, but not the 18-month-old, cohort suggests that the exercise effect on motor function may be triggered initially by increased GDNF expression at an earlier age. However, at a more advanced age given equal exercise regimen, the responsiveness of GDNF expression or signaling may be mitigated. Exercise increases GFRα1 expression, but at 8 months younger than the cohorts in this study [28]. Therefore, the lack of effect of exercise on GFRα1 expression when both age groups reached at least 27 months old (Fig 6), may suggest an age-related decrease in responsiveness of GDNF signaling augmentation in response to exercise. There was also no change in TH expression in the SN from the exercise regimen, (Fig 7B), despite that the same regimen increased TH in the SN if ending 8 months earlier in the lifespan [28]. However, exercise may have increased the number of TH+ neurons in the 18 month old group, and it will be necessary to evaluate whether exercise can increase the number of TH+ neurons to further evaluate exercise impact and possible impact on locomotor function in a future study. Exercise impact on TH phosphorylation is also not reported in this study, but we have previously reported that a shorter duration of this exercise regimen had no effect on ser19 or ser31 phosphorylation in the striatum [28]. However, evaluation of TH phosphorylation in future exercise studies could potentially identify signaling mechanisms activated by exercise, including increased GDNF signaling which increases ser31 TH phosphorylation in the SN [56].

Exogenous GFRα1 delivered into the SN increases TH expression in rats of similar age in this study with increased locomotor activity [30]. Therefore, our results collectively indicate that exercise impact on motor function may be dependent on the plasticity of GDNF signaling, either through augmentation of ligand or receptor expression. As aging reduces GFRα1 expression [29], we speculate that aging itself may be a barrier to exercise impact. Overcoming this barrier may prevent nigral TH loss that is seen in aging rats [36,37], primates [61], and human [62,63]. An evaluation of GFRα1 and TH expression at the point when exercise reverses aging-related motor decline would further support this possibility. Furthermore, quantifying GDNF expression in both striatum and SN in a similar exercise study will be essential to determine if increased GDNF expression, as reported in other exercise studies, [26,27], could trigger changes in TH expression or phosphorylation, and GFRα1 expression in association with exercise impact. As such, an increase in GDNF expression following exercise initiation in the two age groups studied here would further point to whether diminished plasticity of GFRα1 expression to a possible increase in GDNF levels is the critical factor for possible reduced exercise efficacy in aging.

In summary, consistent periods of moderate exercise, interspersed with rest, can reduce aging-related motor decline when initiated at a time in the lifespan when motor decline has already begun. However, given an identical time period of exercise exposure when initiated at an older age in the lifespan, exercise intervention is not similarly effective within the same time frame, suggesting that the window for exercise intervention may rest somewhere within the final 1/3 of the entire lifespan. Our study also suggests the possibility that increasing the frequency of exercise by reducing non-exercise or rest periods, may produce an exercise benefit, despite an older age. Mechanistically, whereas exercise can increase GFRα1 and TH expression in the SN at an earlier period in the lifespan, this study indicates that these increases may be dependent upon increased exercise frequency at old age. Our study also establishes a framework within which to further evaluate critical molecular mechanisms associated with exercise-mediated deceleration of aging-related motor decline.

## Supporting information

S1 Fig **Body weight changes during acclimation to exercise regimen and during treadmill exercise initiation at 18 months (A,C) or 24 months old (B,D).** Results are expressed as percent change in body weight, with respect to each individual’s baseline body weight, during each acclimation period. Data are presented as mean ± SEM. **A. Percent change in body weight during acclimation at 18 months of age.** There was a significant main effect of time (F_(1,18)_ = 42.25, *p*<0.0001) between the two acclimation periods. The percent change in body weight was significantly greater in the second period compared to the first period (****p<0.0001 (non-exercise), ***p<0.00 (exercise)). **B. Percent change in body weight during treadmill acclimation at 24 months of age.** There was a significant main effect of time (F_(1,12)_ = 24.01, *p*<0.001) between the two acclimation periods. The percent change in body weight was significantly greater during the second acclimation period compared to the first acclimation period (**p<0.01 (Non-Exercise), *p<0.05 (Exercise)). **C. Percent change in body weight during each round of the regimen starting at 18 months old.** There was a significant main effect of time (F_(7,63)_ = 4.17, *p*<0.001) and group (F_(1,9)_ = 6.37, *p*<0.05) during treadmill exercise. Percent change in body weight was also significantly greater in exercise compared to non-exercise rats during rounds 3 (*p<0.05) and 7 (**p<0.01), and there was a trend during round 1 (*p* = 0.068). **D. Percent change in body weight during each round of the regimen starting at 24 months old.** There was a significant main effect of time (F_(4,48)_ = 6.17, *p*<0.001) and group (F_(1,12)_ = 6.72, *p*<0.05) during exercise. Post-hoc tests showed that exercise rats had a greater percent change in body weight compared to non-exercise rats during round 3 (**p<0.01).
(PDF)

S2 Fig **Exercise impact at 18 or 24 months on DA tissue content. Striatum (A,B). A. 18 months.** DA tissue content was not significantly affected by the exercise regimen in 18 month old rats (*t* = 1.38, *p* = 0.19). **B. 24 months** DA tissue content was not significantly affected by the exercise regimen in 24 month old rats (*t* = 0.86, *p* = 0.41). **Substantia nigra (C,D) C. 18 months** DA tissue content was not significantly affected by the exercise regimen in 18 month old rats (*t* = 0.12, *p* = 0.90). **D. 24 months** DA tissue content was not significantly affected by the exercise regimen in 24 month old rats (*t* = 0.03, *p* = 0.98). **Nucleus accumbens (E,F) E. 18 months** DA tissue content was not significantly affected by the exercise regimen in 18 month old rats (*t* = 0.76, *p* = 0.46). **F. 24 months** DA tissue content was not significantly affected by the exercise regimen in 24 month old rats (*t* = 1.31, *p* = 0.22). **Ventral tegmental area (G,H). G. 18 months.** There was a trend toward an increase in DA tissue content after exercise (*t* = 1.98, *p* = 0.06). **H. 24 months.** DA tissue content was not significantly affected by the exercise regimen in 24 month old rats (*t* = 0.69, *p* = 0.51).
(PDF)

## Abbreviations

DA: dopamine
DOPAC: dihydroxyphenylacetic acid
PD: Parkinson’s disease
TH: tyrosine hydroxylase
SN: substantia nigra
VTA: ventral tegmental area
NAc: nucleus accumbens
GFRα1: GDNF family receptor

## References

[1] Bennett DA, Beckett LA, Murray AM, Shannon KM, Goetz CG, Pilgrim DM, et al Prevalence of Parkinsonian signs and associated mortality in a community population of older people. New England J Med. 1996;334; 71–76. 8531961 10.1056/NEJM199601113340202

[2] Murray AM, Bennett DA, Mendes de Leon CF, Beckett LA, Evans DA. A longitudinal study of Parkinsonism and disability in a community population of older people. J Gerontol A Biol Sci Med Sci. 2004;59: 864–870. 15345740 10.1093/gerona/59.8.m864

[3] Fleischman DA, Wilson RS, Schneider JA, Beinias JL, Bennet DA. Parkinsonian signs and functional disability in old age. Exp Aging Res. 2007;33: 59–76. doi: 10.1080/03610730601006370 17132564 10.1080/03610730601006370

[4] Buchman AS, Wilson RS, Boyle PA, Bienias JL, Bennett DA. 2007. Change in motor function and risk of mortality in older persons. J Am Geriatr Soc. 2007;55: 11–19. doi: 10.1111/j.1532-5415.2006.01032.x 17233680 10.1111/j.1532-5415.2006.01032.x

[5] Buchman AS, Shulman JM, Nag S, Leurgans S E, Arnold SE, Morris MC, et al Nigral pathology and parkinsonian signs in elders without Parkinson disease. Ann. Neurol. 2012;71: 258–266. doi: 10.1002/ana.22588 22367997 10.1002/ana.22588 PMC3367476

[6] Buchman AS, Leurgans SE, Yu L, Wilson RS, Lim AS, James BD, et al Incident parkinsonism in older adults without Parkinson disease. Neurology. 2016;87: 1036–1044. doi: 10.1212/WNL.0000000000003059 27488597 10.1212/WNL.0000000000003059 PMC5027813

[7] Rosano C, Bennett DA, Newman AB, Venkatraman V, Yaffe K, Harris T, et al Patterns of focal gray matter atrophy are associated with bradykinesia and gait disturbances in older adults. J Gerontol A Biol Sci Med Sci. 2012; 67: 957–962. doi: 10.1093/gerona/glr262 22367436 10.1093/gerona/glr262 PMC3436092

[8] U.S. Department of Health and Human Services, Living Long & Well in the 21st Century Strategic Directions for Research on Aging. 2007 National Institue on Aging. NIH Publication Number 07–6252.

[9] Savela SL, Koistinen P, Tilvis RS, Strandberg AY, Pitkala KH, Salomaa VV, et al 2010. Physical activity at midlife and health-related quality of life in older men. Arch Intern Med 2010;170: 1171–1172. doi: 10.1001/archinternmed.2010.187 20625028 10.1001/archinternmed.2010.187

[10] Cooper R, Mishra GD, Kuh D. Physical activity across adulthood and physical performance in midlife: findings from a British birth cohort. Am. J. Prev. Med. 2011;41: 376–384. doi: 10.1016/j.amepre.2011.06.035 21961464 10.1016/j.amepre.2011.06.035 PMC3185208

[11] Stenholm S, Koster A, Valkeinen H, Patel KV, Bandinelli S, Guralnik JM, et al Association of Physical Activity History With Physical Function and Mortality in Old Age. J Gerontol A Biol Sci Med Sci. 2016; doi: 10.1093/Gerona/glv111 26290538 10.1093/gerona/glv111 PMC4834834

[12] Herman T, Giladi N, Gruendlinger L, Hausdorff JM. Six weeks of intensive treadmill training improves gait and quality of life in patients with Parkinson’s disease: A pilot study. Arch Phys Med Rehabil 2007;88: 1154–1158. doi: 10.1016/j.apmr.2007.05.015 17826461 10.1016/j.apmr.2007.05.015

[13] Ridgel AL, Vitek JL, Alberts JL. Forced, not voluntary, exercise improves motor function in Parkinson’s disease patients. Neurorehabiol Neural Repair. 2009;23: 600–608. 10.1177/1545968308328726 19131578

[14] Nadeau A, Pourcher E, Corbeil P. Effects of 24 wk of treadmill training on gait performance in Parkinson’s disease. Med Sci Sports Exerc. 2014;46: 645–655. doi: 10.1249/MSS.0000000000000144 24002341 10.1249/MSS.0000000000000144

[15] Shen X, Wong-Yu IS, Mak MK. Effect of exercise on falls, balnce, and gait ability in Parkinson’s disease: A meta-analysis. Neurorehabil Neural Repair 2016; 30: 512–527. doi: 10.1177/1545968315613447 26493731 10.1177/1545968315613447

[16] Smith AD, Zigmond MJ. Can the brain be protected through exercise? Lessons from an animal model of parkinsonism. Exp Neurol. 2003;184: 31–39. 14637076 10.1016/j.expneurol.2003.08.017

[17] Tillerson JL, Caudle WM, Reveron ME, Miller GW. Exercise induces behavioral recovery and attenuates neurochemical deficits in rodent models of Parkinson’s disease. Neuroscience 20003;119: 899–911. 10.1016/s0306-4522(03)00096-4 12809709

[18] Petzinger GM, Walsh JP, Akopian G, Hogg E, Abernathy A, Arevalo P, et al Effects of treadmill exercise on dopaminergic transmission in the 1-methyl-4-phenyl-1,2,3,6-tetrahydropyridine-lesioned mouse model of basal ganglia injury. J Neurosci. 2007;27: 5291–5300. doi: 10.1523/JNEUROSCI.1069-07.2007 17507552 10.1523/JNEUROSCI.1069-07.2007 PMC6672356

[19] Smith BA, Goldberg NR, Medhul CK. Effects of treadmill exercise on behavioral recovery and neural changes in the substantia nigra and striatum of the 1-methyl-4-phenyl-1,2,3,6-tetrahydropyridine-lesioned mouse. Brain Res. 2011;1386: 70–80. doi: 10.1016/j.brainres.2011.02.003 21315689 10.1016/j.brainres.2011.02.003 PMC3073026

[20] Hillsdon MM, Brunner EJ, Guralnik JM, Marmot MG. Prospective study of physical activity and physical function in early old age. Am J Prev Med. 2005;28: 245–250. doi: 10.1016/j.amepre.2004.12.008 15766611 10.1016/j.amepre.2004.12.008

[21] Denison HJ, Syddall HE, Dodds R, Martin HJ, Finucane FM, Griffin SJ, et al Effects of aerobic exercise on muscle strength and physical performance among community dwelling older people from the Hertfordshire Cohort Study: A randomized controlled trial. J Am Geriatr Soc. 2013;61: 1034–1036. doi: 10.1111/jgs.12286 23772733 10.1111/jgs.12286 PMC3708295

[22] Santos D, Mahoney JR, Allali G, Verghese J. Physical activity in older adults with mild Parkinsonian signs: A cohort study. J Gerontol A Biol Sci. 2017; doi: 10.1093/gerona/glx133 10.1093/gerona/glx133 PMC6230212 29931236

[23] Prince SA, Adamo KB, Hamel ME, Hardt J, Gorber SC, Tremblay M. A comparison of direct versus self-report measures for assessing physical activity in adults: a systematic review. Int J Behav Nutr Phys Act. 2008;5: 56 doi: 10.1186/1479-5868-5-56 18990237 10.1186/1479-5868-5-56 PMC2588639

[24] Hoffer BJ, Hoffman AF, Bowenkamp KE, Huettl P, Hudson J, Martin D, et al Glial cell line-derived neurotrophic factor reverses toxin-induced injury to midbrain dopaminergic neurons in vivo. Neurosci Lett. 1994;182: 107–111. 7891873 10.1016/0304-3940(94)90218-6

[25] Grondin R., Cass WA, Zhang Z, Stanford JA, Gash DM, Gerhardt GA. Glial cell line-derived neurotrophic factor increases stimulus-evoked dopamine release and motor speed in aged rhesus monkeys. J Neurosci. 2003; 23: 1974–1980. 12629203 10.1523/JNEUROSCI.23-05-01974.2003 PMC6741972

[26] Tajari N, Yasuhara T, Shingo T, Kondo A, Yuan W, Kadota T, et al Exercise exerts neuroprotective effects on Parkinson's disease model of rats. Brain Res. 2010; 1310: 200–207. doi: 10.1016/j.brainres.2009.10.075 19900418 10.1016/j.brainres.2009.10.075

[27] McCullough MJ, Gyorkos AM, Spitsbergen JM. Short-term exercise increases GDNF protein levels in the spinal cord of young and old rats. Neuroscience. 2013; 240: 258–268. doi: 10.1016/j.neuroscience.2013.02.063 23500094 10.1016/j.neuroscience.2013.02.063 PMC3637874

[28] Arnold JC, Salvatore MF. Exercise-mediated increase in nigral tyrosine hydroxylase is accompanied by increased nigral GFR-α1 and EAAC1 expression in aging rats. ACS Chem. Neurosci. 2016;7: 227–239. doi: 10.1021/acschemneuro.5b00282 26599339 10.1021/acschemneuro.5b00282 PMC4926611

[29] Pruett BS, Salvatore MF. GFRα-1 receptor expression in the aging nigrostriatal and mesoaccumbens pathways. J Neurochem. 2010; 115: 707–715. doi: 10.1111/j.1471-4159.2010.06963.x 20731758 10.1111/j.1471-4159.2010.06963.x

[30] Pruett BS, Salvatore MF. Nigral GFRα-1 infusion in aged rats increases locomotor activity, nigral tyrosine hydroxylase, and dopamine content in synchronicity. Mol Neurobiol. 2013; 47: 988–999. doi: 10.1007/s12035-013-8397-7 23321789 10.1007/s12035-013-8397-7 PMC4037235

[31] Salvatore MF, Terrebonne J, Fields V, Nodurft D, Runfalo C, Latimer B, Ingram DK. 2016. Initiation of calorie restriction in middle-aged male rats attenuates aging-related motoric decline and bradykinesia without increased striatal dopamine. Neurobiol. Aging 2016;37: 192–207. doi: 10.1016/j.neurobiolaging.2015.10.006 26610387 10.1016/j.neurobiolaging.2015.10.006 PMC4688216

[32] Salvatore MF, Terrebonne J, Cantu MA, McInnis TR, Venable K, Kelley P, et al Dissociation of striatal dopamine and tyrosine hydroxylase expression from aging-related motor decline: evidence from calorie restriction intervention. J Gerontol A Biol Sci. 2017; doi: 10.1093/gerona/glx119 28637176 10.1093/gerona/glx119 PMC5861909

[33] Arnold JC, Salvatore MF. Getting to compliance in forced exercise in rodents: a critical standard to evaluate exercise impact in aging-related disorders and disease. J Vis Exp. 2014 90: e51827. 10.3791/51827 PMC4207632 25178094

[34] Batra A, Coxe S, Page TE, Melchior M, Palmer RC. Evaluating the factors associated with the completion of a community-based group exercise program among older women. J Aging Phys Act. 2016;24: 649–658. doi: 10.1123/japa.2015-0281 27122546 10.1123/japa.2015-0281

[35] Hawley-Hague H, Horne M, Skelton DA, Tood C. Older adults uptake and adherence to exercise classes: Instructor’s perspectives. J Aging Phy Act. 2016; 24: 649–658. 10.1123/japa.2014-0108 26214265

[36] Salvatore MF, Pruett BS, Spann SL, Dempsey C. Aging reveals a role for nigral tyrosine hydroxylase ser31 phosphorylation in locomotor activity generation. PLoS ONE 2009;4(12): e8466 doi: 10.1371/journal.pone.0008466 20037632 10.1371/journal.pone.0008466 PMC2791868

[37] Yurek M, Hipkens SB, Hebert MA, Gash DM, Gerhardt GA. 1998. Age-related decline in striatal dopamine release and motoric function in Brown Norway/Fischer 344 hybrid rats. Brain Res. 1998; 791: 246–256. 9593919 10.1016/s0006-8993(98)00110-3

[38] Lushaj EB, Johson JK, McKenzie D, Aiken JM. Sarcopenia accelerates at advanced ages in Fisher344xBrown Norway Rats. J Gerontol A Biol Sci Med Sci. 2008;63: 921–927. 18840796 10.1093/gerona/63.9.921 PMC2902273

[39] Paratcha G, Ledda F, Baars L, Coulpier M, Besset V, Anders J, et al Released GFRalpha1 potentiates downstream signaling, neuronal survival, and differentiation via a novel mechanism of recruitment of c-Ret to lipid rafts. Neuron. 2001;29: 171–184. 11182089 10.1016/s0896-6273(01)00188-x

[40] Matsuo A, Nakamura S, Akiguchi I. Immunohistochemical localization of glial cell line-derived neurotrophic factor family receptor GFR in the rat brain: confirmation of expression in various neuronal systems. Brain Res. 2000;859: 57–71. 10720615 10.1016/s0006-8993(99)02442-7

[41] Harvey JA, Chastin SF, Skelton DA. Prevalence of sedentary behavior in older adults: a systematic review. Int J Environ Res Public Health 2013;10: 6645–6661. doi: 10.3390/ijerph10126645 24317382 10.3390/ijerph10126645 PMC3881132

[42] Patel KV, Coppin AK, Manini TM, Lauretani F, Bandinelli S, Ferrucci L, et al Midlife physical activity and mobility in older age: The InCHIANTI study. Am J Prev Med. 2006;31: 217–224. doi: 10.1016/j.amepre.2006.05.005 16905032 10.1016/j.amepre.2006.05.005 PMC2646092

[43] Musich S, Wang SS, Hawkins K, Greame C. The frequency and health benefits of physical activity in older adults. Popul Health Manag. 2017; 20: 199–207. doi: 10.1089/pop.2016.0071 27623484 10.1089/pop.2016.0071 PMC5488312

[44] Skalicky M, Bubna-Littitz H, Viidik A. Influence of physical exercise on aging rats: I. Life-long exercise preserves patterns of sponateous activity. Mech Age Dev. 1996;87: 127–139. 10.1016/0047-6374(96)01707-1 8783195

[45] Viidik A, Skalicky M. Influence of physical exercise on old rats: Changes in patterns of spontaneous activity and connective tissues. Aging Clin Exp Res. 1997;9: 64–72. 10.1007/BF03340129 9177587

[46] Wong-Yu IS, Mak MK. Multi-dimensional balance training programme improves balane and gait performance in people with Parkinson’s disease: A pragmatic randomized controlled trial with 12-month follow-up. Parkinsonism Relat Disord 2015: 21: 615–621. doi: 10.1016/j.parkreldis.2015.03.022 25899544 10.1016/j.parkreldis.2015.03.022

[47] Carvalho A, Barbirato D, Araujo N, Martins JV, Cavalcanti JL, Santos TM, et al Comparison of strength training, aerobic training, and additional physical therapy as supplementary treatments for Parkinson’s disease: pilot study. Clin Interv Aging. 2015;10: 183–191. doi: 10.2147/CIA.S68779 25609935 10.2147/CIA.S68779 PMC4293290

[48] Peacock CA, Sanders GJ, Wilson KA, Fickes-Ryan EJ, Corbett DB, von Carlowitz KP, et al Introducing a multifaceted exercise intervention particular to older adults diagnosed with Parkinson’s disease: a preliminary study. Aging Clin Exp Res. 2014; 26: 403–409. doi: 10.1007/s40520-013-0189-4 24347123 10.1007/s40520-013-0189-4

[49] Abercrombie ED, Zigmond MJ. Chapter 31: Modification of central categcholaminergic systems by stress and injury: Functional significance and clinical implications In: Bloom F.E, Kupfer D.J, editors. Psychopharmacology: The Fourth Generation of Progress. Vol. 31. New York: Raven Press Ltd; 1995 pp. 355–361.

[50] Ong LK, Guan L, Stutz B, Dickson PW, Dunkley PR, Bobrovskaya L. The effects of footshock and immobilization stress on tyrosine hydroxylase phosphorylation in the rat locus coeruleus and adrenal gland. Neuroscience. 2011; 192: 20–27. doi: 10.1016/j.neuroscience.2011.06.087 21767616 10.1016/j.neuroscience.2011.06.087

[51] Seldeen KL, Lasky G, Leiker MM, Pang M, Personius KE, Troen BR. High intensity interval training improves physical performance and frailty in aged mice. J Gerontol A Biol Sci. 2017; doi: 10.1093/gerona/glx120 28633487 10.1093/gerona/glx120

[52] Mänty M, Kuh D, Cooper R. Associations of midlife to late life fatigue with physical performance and strength in early old age: Results from a British Prospective Cohort Study. Psychosom. Med. 2015;77: 823–832. doi: 10.1097/PSY.0000000000000214 26176776 10.1097/PSY.0000000000000214 PMC4568292

[53] Egerton T, Chastin SF, Stensvold D, Helbostad JL. Fatigue may contribute to reduced physical activity among older people: An observational study. J. Gerontol. A. Biol. Sci. Med Sci. 2016;71: 670–676. doi: 10.1093/gerona/glv150 26347508 10.1093/gerona/glv150

[54] Keysor JJ, Jette AM. Have we oversold the benefit of late-life exercise? J Gerontol A Biol Sci. Med. Sci. 2001;56: M412–423. 11445600 10.1093/gerona/56.7.m412

[55] VanSwearingen JM, Perera S, Brach JS, Wert D, Studenski SA. Impact of exercise to improve gait efficiency on activity and participation in older adults with mobility limitations: a randomized controlled trial. Phys Ther. 2011;91: 1740–1751. doi: 10.2522/ptj.20100391 22003158 10.2522/ptj.20100391 PMC3229041

[56] Manini TM, Pahor M. Physical activity and maintaining physical function in older adults. Br J Sports Med 2016;43: 28–31. 10.1136/bjsm.2008.053736 PMC3104323 18927164

[57] Haycock JW, Becker L, Ang L, Furukawa Y, Hornykiewicz O, Kish SJ. Marked disparity between age related changes in dopamine and other presynaptic dopaminergic markers in human striatum. J Neurochem. 2003;87: 574–585. 14535941 10.1046/j.1471-4159.2003.02017.x

[58] Wolf ME, LeWitt PA, Bannon MJ, Dragovic LJ, Kapatos G. Effect of aging on tyrosine hydroxylase protein content and the relative number of dopamine nerve terminals in human caudate. J Neurochem. 1991;56: 1191–1200. 1672141 10.1111/j.1471-4159.1991.tb11410.x

[59] Salvatore M.F., Zhang J.L., Large D.M., Wilson P.E., Thomas T.D., Gash C.R., et al Striatal GDNF administration increases tyrosine hydroxylase phosphorylation in the rat striatum and substantia nigra. J. Neurochem. 2004;90: 245–254. doi: 10.1111/j.1471-4159.2004.02496.x 15198683 10.1111/j.1471-4159.2004.02496.x

[60] Maswood N, Young J, Tilmont E, Zhang Z, Gash DM, Gerhardt GA, et al Caloric restriction increases neurotrophic factor levels and attenuates neurochemical and behavioral deficits in a primate model of Parkinson's disease. Proc Nat Acad Sci 2004;101: 18171–18176. doi: 10.1073/pnas.0405831102 15604149 10.1073/pnas.0405831102 PMC539733

[61] Emborg ME, Ma SY, Mufson EJ, Levey AI, Taylor MD, Brown WD, et al Age-related declines in nigral neuronal function correlate with motor impairments in rhesus monkeys. J. Comp. Neurol. 1998;401: 253–265. 9822152

[62] Fearnley JM, Less AJ. 1991 Ageing and Parkinson’s disease: substantia nigra regional selectivity. Brain 1991; 114(Pt 5): 2283–2301. 1933245 10.1093/brain/114.5.2283

[63] Ross GW, Petrovich H, Abbott RD, Nelson J, Markesbery W, Davis D, et al Parkinsonian signs and substantia nigra neuron density in descendants elders without PD. Ann Neurol. 2004;56: 532–539. doi: 10.1002/ana.20226 15389895 10.1002/ana.20226

