# Supplementary material for: Aging-related limit of exercise efficacy on motor decline
Source: PLoS One. 2017 Nov 27;12(11):e0188538. doi: 10.1371/journal.pone.0188538 (PMC5703560; doi:10.1371/journal.pone.0188538)
Supplement: S1 Fig — Body weight changes during acclimation to exercise regimen and during treadmill exercise initiation at 18 months (A,C) or 24 months old (B,D). Results are expressed as percent change in body weight, with respect to each individual’s baseline body weight, during each acclimation period. Data are presented as mean ± SEM. A. Percent change in body weight during acclimation at 18 months of age. There was a significant main effect of time (F(1,18) = 42.25, p<0.0001) between the two acclimation periods. The percent change in body weight was significantly greater in the second period compared to the first period (****p<0.0001 (non-exercise), ***p<0.00 (exercise)). B. Percent change in body weight during treadmill acclimation at 24 months of age. There was a significant main effect of time (F(1,12) = 24.01, p<0.001) between the two acclimation periods. The percent change in body weight was significantly greater during the second acclimation period compared to the first acclimation period (**p<0.01 (Non-Exercise), *p<0.05 (Exercise)). C. Percent change in body weight during each round of the regimen starting at 18 months old. There was a significant main effect of time (F(7,63) = 4.17, p<0.001) and group (F(1,9) = 6.37, p<0.05) during treadmill exercise. Percent change in body weight was also significantly greater in exercise compared to non-exercise rats during rounds 3 (*p<0.05) and 7 (**p<0.01), and there was a trend during round 1 (p = 0.068). D. Percent change in body weight during each round of the regimen starting at 24 months old. There was a significant main effect of time (F(4,48) = 6.17, p<0.001) and group (F(1,12) = 6.72, p<0.05) during exercise. Post-hoc tests showed that exercise rats had a greater percent change in body weight compared to non-exercise rats during round 3 (**p<0.01). (PDF) [file pone.0188538.s001.pdf]

Supplemental Figure 1

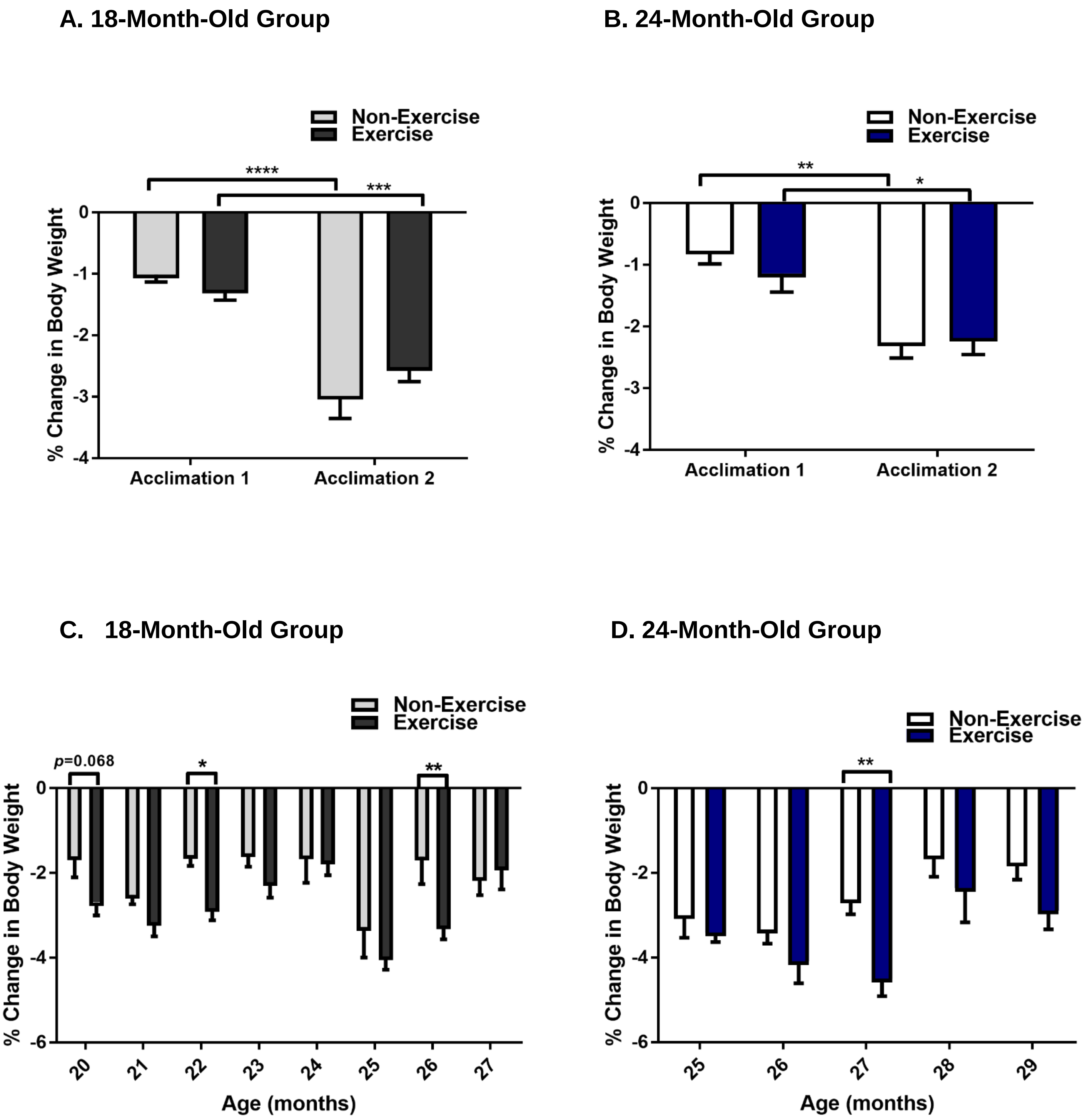

Supplemental Figure 2. Body weight changes during acclimation to exercise regimen and during treadmill exercise initiation at 18 months (A,C) or 24 months old (B,D). Results are expressed as percent change in body weight, with respect to each individual's baseline body weight, during each acclimation period. Data are presented as mean ± SEM. **A. Percent change in body weight during acclimation at 18 months of age.** There was a significant main effect of time ($F_{(1,18)}$= 42.25, $p<0.0001$) between the two acclimation periods. The percent change in body weight was significantly greater in the second period compared to the first period (****$p<0.0001$ (non-exercise), ***$p<0.001$ (exercise)). **B. Percent change in body weight during treadmill acclimation at 24 months of age.** There was a significant main effect of time ($F_{(1,12)}$ = 24.01, $p<0.001$) between the two acclimation periods. The percent change in body weight was significantly greater during the second acclimation period compared to the first acclimation period (**$p<0.01$ (Non-Exercise), *$p<0.05$ (Exercise)). **C. Percent change in body weight during each round of the regimen starting at 18 months old.** There was a significant main effect of time ($F_{(7,63)}$= 4.17, $p<0.001$) and group ($F_{(1,9)}$=6.37, $p<0.05$) during treadmill exercise. Percent change in body weight was also significantly greater in exercise compared to non-exercise rats during rounds 3 (*$p<0.05$) and 7 (**$p<0.01$), and there was a trend during round 1 (p=0.068). **D. Percent change in body weight during each round of the regimen starting at 24 months old.** There was a significant main effect of time ($F_{(4,48)}$= 6.17, $p<0.001$) and group ($F_{(1,12)}$=6.72, $p<0.05$) during exercise. Post-hoc tests showed that exercise rats had a greater percent change in body weight compared to non-exercise rats during round 3 (**$p<0.01$).
