# Supplementary material for: Aging-related limit of exercise efficacy on motor decline
Source: PLoS One. 2017 Nov 27;12(11):e0188538. doi: 10.1371/journal.pone.0188538 (PMC5703560; doi:10.1371/journal.pone.0188538)
Supplement: S2 Fig — Exercise impact at 18 or 24 months on DA tissue content. Striatum (A,B). A. 18 months. DA tissue content was not significantly affected by the exercise regimen in 18 month old rats (t = 1.38, p = 0.19). B. 24 months DA tissue content was not significantly affected by the exercise regimen in 24 month old rats (t = 0.86, p = 0.41). Substantia nigra (C,D) C. 18 months DA tissue content was not significantly affected by the exercise regimen in 18 month old rats (t = 0.12, p = 0.90). D. 24 months DA tissue content was not significantly affected by the exercise regimen in 24 month old rats (t = 0.03, p = 0.98). Nucleus accumbens (E,F) E. 18 months DA tissue content was not significantly affected by the exercise regimen in 18 month old rats (t = 0.76, p = 0.46). F. 24 months DA tissue content was not significantly affected by the exercise regimen in 24 month old rats (t = 1.31, p = 0.22). Ventral tegmental area (G,H). G. 18 months. There was a trend toward an increase in DA tissue content after exercise (t = 1.98, p = 0.06). H. 24 months. DA tissue content was not significantly affected by the exercise regimen in 24 month old rats (t = 0.69, p = 0.51). (PDF) [file pone.0188538.s002.pdf]

Supplemental Figure 2

A. striatum (18 mo)

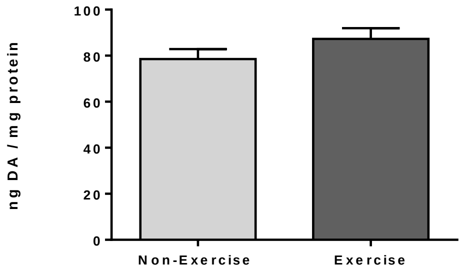

B. striatum (24 mo)

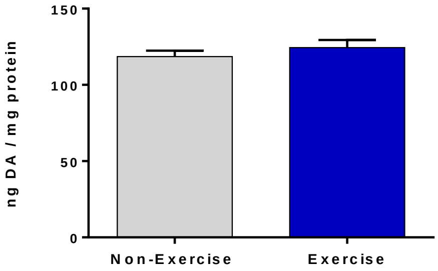

C. SN (18 mo)

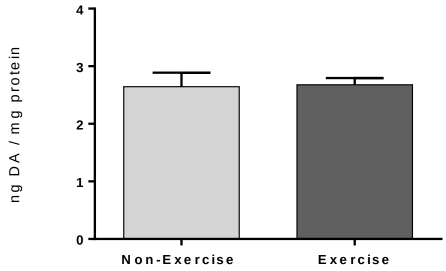

D. SN (24 mo)

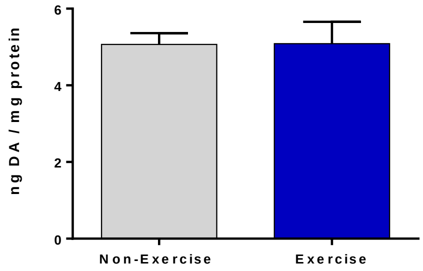

E. NAc (18 mo)

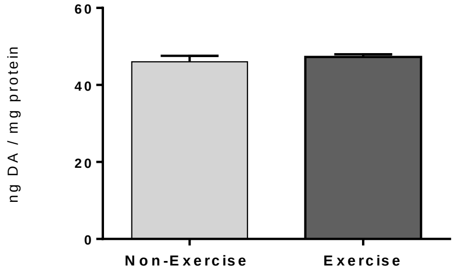

F. NAc (24 mo)

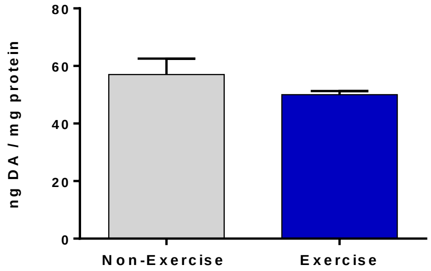

G. VTA (18 mo)

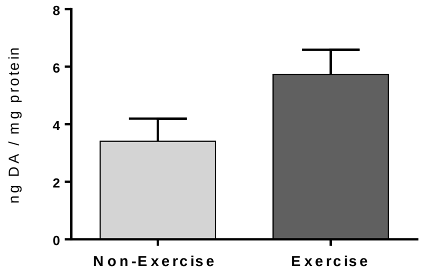

H. VTA (mo)

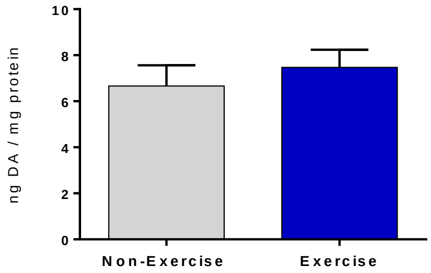

Supplemental Figure 2. Exercise impact at 18 or 24 months on DA tissue content. Striatum (A,B). A. 18 months. DA tissue content was not significantly affected by the exercise regimen in 18 month old rats (t=1.38, p=0.19). **B. 24 months** DA tissue content was not significantly affected by the exercise regimen in 24 month old rats (t=0.86, p=0.41). **Substantia nigra (C,D) C. 18 months** DA tissue content was not significantly affected by the exercise regimen in 18 month old rats (t=0.12, p=0.90). **D. 24 months** DA tissue content was not significantly affected by the exercise regimen in 24 month old rats (t=0.03, p=0.98). **Nucleus accumbens (E,F) E. 18 months** DA tissue content was not significantly affected by the exercise regimen in 18 month old rats (t=0.76, p=0.46). **F. 24 months** DA tissue content was not significantly affected by the exercise regimen in 24 month old rats (t=1.31, p=0.22). **Ventral tegmental area (G,H). G. 18 months.** There was a trend toward an increase in DA tissue content after exercise (t=1.98, p=0.06). **H. 24 months.** DA tissue content was not significantly affected by the exercise regimen in 24 month old rats (t=0.69, p=0.51).
